# Concurrent chemoradiotherapy followed by adjuvant chemotherapy versus concurrent chemoradiotherapy alone in locally advanced cervical cancer: A systematic review and meta-analysis

**DOI:** 10.3389/fonc.2022.997030

**Published:** 2022-12-07

**Authors:** Haonan Liu, Xiao Ma, Chenyu Sun, Meng Wu, Zhiyuan Xu, Shuang Zhou, Nan Yao, Suya Liu, Xiaobing Qin, Zhengxiang Han

**Affiliations:** ^1^ Department of Oncology, The Affiliated Hospital of Xuzhou Medical University, Jiangsu, China; ^2^ Department of Internal Medicine, AMITA Health Saint Joseph Hospital Chicago, Chicago, IL, United States; ^3^ Department of Emergency, The Affiliated Hospital of Xuzhou Medical University, Jiangsu, China

**Keywords:** concurrent, chemoradiotherapy, adjuvant chemotherapy, cervical cancer, meta-analysis

## Abstract

**Objective:**

This study aimed to assess the efficacy and safety of adjuvant chemotherapy (ACT) after concurrent chemoradiation (CCRT) in patients with locally advanced cervical cancer (LACC) *via* meta-analysis.

**Methods:**

A systematic literature search of MEDLINE, PubMed, Web of Science, EMBASE, and the Cochrane Central Register of Controlled Trials was conducted from January 10, 1966 to May 20, 2022. Randomized controlled trials and observational studies comparing the CCRT alone with CCRT plus ACT were included. The literature search, quality assessment, and data extraction were conducted by two reviewers independently. The primary endpoints were 3-year rates of overall survival (OS) and progression-free survival (PFS). Complete response rate, local recurrence, distant metastasis, and adverse events were secondary outcomes. The hazard ratios (HRs) and relative risk (RR) were pooled.

**Results:**

Nine studies with a total of 2732 patients were included in this meta-analysis, including 1411 patients in the CCRT group and 1321 in the CCRT plus ACT group. The HR for 3-year rates of OS and PFS of the CCRT group compared with the CCRT plus ACT group was 0.72 [95%confidence interval (CI) = 0.44–1.17] and 0.78 (95%CI = 0.5–1.75), respectively. No significant differences were observed between the two groups in the complete response rate (RR = 1.06, 95%CI = 0.96–1.16). However, local recurrence and distant metastasis were significantly lower in the CCRT plus ACT group than in the CCRT group (RR = 0.63, 95%CI = 0.44 –0.91 and RR = 0.64, 95%CI = 0.47–0.88). Grade 3–4 acute toxicities were more frequent in the CCRT plus ACT group (RR = 1.73, 95%CI =1.19–2.52).

**Conclusion:**

Although associated with a decreased risk of local recurrence and distant metastasis, ACT did not significantly improve the survival rate and the complete response rate with increasing grade 3–4 acute toxicities in patients with LACC. Thus, this ACT regimen cannot be recommended for patients with LACC.

**Systematic review registration:**

https://inplasy.com/inplasy-2022-9-0089/, identifier INPLASY202290089.

## Introduction

As the most common gynecologic malignant neoplasm reported in women worldwide, the treatment of cervical cancer remains a challenge due to the lack of health infrastructure. In 2018, there were about 36,000 new cases, with 311,365 cancer-related deaths (1). In many developing countries, patients were diagnosed with cervical cancer at at a locally advanced stage, indicating a poor outcome (2).

For more than two decades, cisplatin-based concurrent chemoradiotherapy has been used as a standard therapeutic regimen for locally advanced cervical cancer (LACC), based on the survival benefit and clinical experience (3–7). Despite the use of concurrent chemotherapy, about 16%–60% of patients with LACC still suffer from tumor recurrence or distant metastasis (8). The mortality rate in patients with LACC remains high, with a 5-year survival rate less than 60% (9). Previous studies found that concurrent chemoradiation (CCRT) may improve the 5-year survival rate by 9%–18% (10). Adjuvant chemotherapy (ACT) after CCRT is another option for patients with LACC. ACT aims at decreasing both the mortality rate and the risk of recurrence by eliminating residual malignant tissues outside the radiotherapy target region and treating occult disease in the pelvis. While the role of additional chemotherapy after CCRT for treating LACC has been explored in many studies (11–15), survival benefits after the addition of ACT to CCRT in patients with LACC remain controversial. With limited data from only two trials, a Cochrane review published in 2014 could not find sufficient evidence to support the use of ACT after CCRT and failed to perform meta-analysis (9). However, a number of original studies have been published since then, which were incorporated to evaluate the efficacy and safety of ACT in patients with LACC through meta-analysis.

## Methods

This meta-analysis was registered on INPLASY website (INPLASY202290089), doi:10.37766/inplasy2022.9.0089.

### Search strategy

We conducted a systematic literature search of MEDLINE, PubMed, Web of Science, EMBASE, and the Cochrane Central Register of Controlled Trials, using the following search terms: (concurrent or chemoradiotherapy or chemoradiation or concurrent chemoradiation or concurrent chemoradiotherapy or adjuvant chemotherapy or addition or chemotherapy or consolidation chemotherapy) and (cervical cancer or uterine cervical neoplasm or uterine cervical cancer or cervical). In addition, we supplemented the search by manually reviewing the reference lists of retrieved articles and relevant reviews and by contacting content experts for additional published or unpublished trials.

### Study selection

Two of the authors (Wu and Yao) carried out a preliminary search, scanning all titles for eligibility according to the predefined inclusion criterion. Duplicate publications or datasets were removed. Each title and abstract were reviewed to determine eligibility. After obtaining full abstracts for potentially eligible studies, two reviewers (Qin and Han) worked independently to assess eligibility. A study was considered ineligible from a review of the title and its abstract. In all other cases, the full study was reviewed.

### Inclusion and exclusion criteria

Studies were considered eligible for meta-analysis if they met the following criteria:

(1) patients diagnosed with LACC of the FIGO (International Federation of Gynecology and Obstetrics) stage IB–IVA with at least one measurable lesion and Karnofsky performance score of 70 (16) (2); randomized controlled trials (RCTs) or observational studies (3); all patients aged 18 years or older who had not been previously treated with immunotherapy (4); all study protocols approved by the institutional ethics committee and performed in accordance with the Declaration of Helsinki (5); at least 30 patients included in the study (6); survival rate and complete response rate as the outcomes of interest; and (7) risk estimates with 95% confidence interval (CI) or data to calculate them.

The major exclusion criteria were as follows (1): patients with other malignant tumors (2); the publication in the format of an abstract, comment, or review; and (3) no sufficient data.

### Data extraction

Two authors (Zhou and Sun) independently extracted data using a standardized data-collection form. The following information was recorded: the first author’s name, year of publication, sample size, population demographics, study design, trial length, and country of origin. Our primary efficacy endpoint was the survival rate. Secondary endpoints included complete response rate, local recurrence, distant metastasis, and adverse events. Disagreements were resolved by discussion with the third author (Han). The quality assessment of the RCTs was evaluated using the Cochrane Handbook of 6.2 (17).

### Statistical analysis

We evaluated the efficacy and safety of ACT after CCRT in patients with LACC. Qin and Liu performed all statistical analyses. The hazard ratio (HR) and the 95% CI were used to assess the survival rate of patients with LACC who underwent ACT after CCRT. Because of the lack of information on HR, the estimation of data from the Kaplan–Meier curves were used (18, 19). The risk ratio (RR) was used as the summary statistic for statistical analyses of dichotomous variables. The homogeneity of effect size across studies was tested using Q statistics at the statistically significant level of *P* < 0.10. The *I^2^* statistic, which is a quantitative measure of inconsistency across studies (20), was also calculated. We further conducted the sensitivity analysis to explore the possible explanations for heterogeneity and to examine the influence of various exclusion criteria on the overall risk estimate. Finally, potential publication bias was assessed using Begg’s funnel plots and Egger’s regression test (21, 22). All analyses were carried out using Stata 12.0. P value < 0.05 was considered to be statistically. All data analyses were performed according to the PRISMA statement (23).

## Results

### Literature search

**Figure 1:**
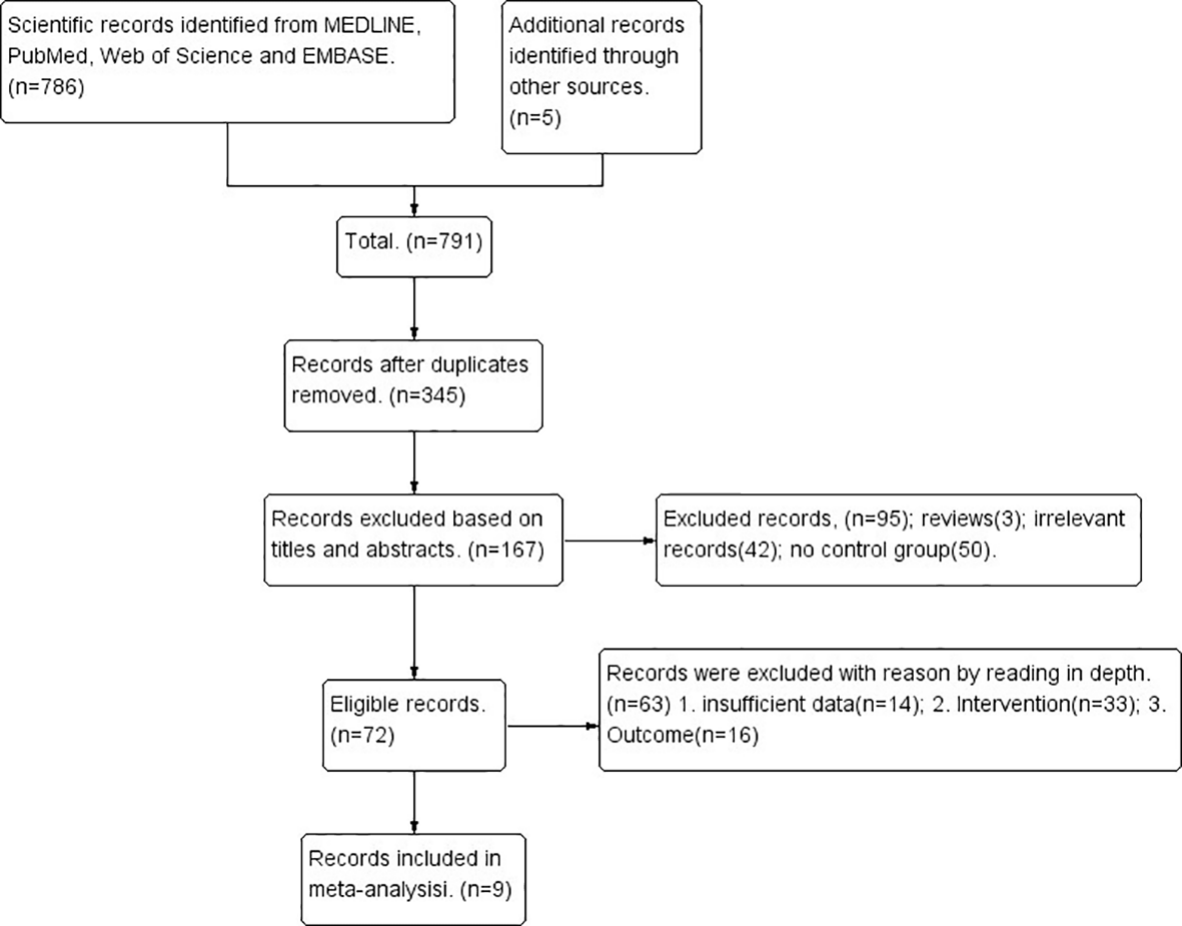
PRISMA flow diagram showing a selection of articles for meta-analysis.

Initially, 791 unique citations were identified. After the removal of duplicates, 345 studies remained eligible. By screening the titles and abstracts, 178 of 345 studies were excluded and 167 were selected for further assessment. Of these publications, 63 studies were excluded for the following reasons: 33 studies did not meet the selection criteria, 14 studies did not provide sufficient data, and 16 studies reported different outcomes. Finally, 9 studies involving a total of 2732 patients (1321 in the CCRT plus ACT group and 1411 in the CCRT-alone group) were included in this meta-analysis. The search process and strategy adopted for this study are shown in **Figure 1**.

### Study characteristics

The characteristics of the included studies are listed in **Table 1**, which were published between 2003 and 2019. Four studies were on RCTs, while the remaining five were observational studies. Of these, two studies were conducted in Thailand (25, 29), two in Korea (27, 30), one in Mexico (28), one in Turkey (24), one in Japan (32), one in Brazil (31), and one in China (26). The median length of the follow-up period ranged from 21.5 to 89 months. This study analyzed 2732 patients with FIGO stage IB–IVA cervical cancer, with the majority of the studies (8/9) using cisplatin-based chemoradiotherapy.

**Table 1:** Details of the previous studies included in this meta-analysis.

Author/year	Country	Research type	Sample size	Follow-up time(median)	Stage	Histopathology	Concurrent chemotherapy	Adjuvant chemotherapy	NOS score
Yavas (2019) (24)	Turkey	Observational study	109	24.5 months	IB to IVA	SCC,ACA,AS,small-cell, large-cell	Cisplatin in both arms	Paclitaxel/carboplatin median 6 cycles (range 3–6 cycles)	6
Tangjit (2019) (25)	Thailand	RCT	259	27.4 months	IIB to IVA	SCC, ACA, AS	Cisplatin in both arms	Paclitaxel/carboplatin 3 cycles	–
Tang (2012) (26)	China	RCT	880	60 months	IIB to IVA	ACA only	Cisplatin in both arms	Paclitaxel/cisplatin 2 cycles	–
Choi (2011) (27)	Korea	Observational study	78	35 months	IIB to IVA	SCC, ACA	5-FU and cisplatin or cisplatin in both arms	5-FU and cisplatin 3 additional cycles	8
Duenas (2011) (28)	Mexico	RCT	515	46.9 months	IIB to IVA	SCC, ACA, AS	Cisplatin in CCRT arm Cisplatin/gemcitabine in CCRT+ACT arm	Cisplatin/gemcitabine 2 cycles	–
Lordvith (2003) (29)	Thailand	RCT	463	89 months	IIB to IVA	SCC, ACA	Mitomycin/oral 5-FU in both arms	Oral 5-FU 3 cycles	–
Kim (2007) (30)	Korea	Observational study	205	64 months	IB to IIB	SCC, small-cell, large-cell	Cisplatin/carboplatin in both arms	Cisplatin/carboplatin 3 cycles	8
Fabri (2019) (31)	Brazil	Observational study	186	37.7 months	IB2,IIA2, or IIB to IVB	SCC, ACA	Cisplatin in both arms	cisplatin and gemcitabine 2 cycles	7
Abe (2011) (32)	Japan	Observational study	37	21.5 months	IB to IVA	SCC, ACA	Cisplatin in both arms	carboplatin and paclitaxel for 3-6 cycles	7

ACA, adenocarcinoma; ACT, adjuvant chemotherapy; AS, adenosquamous carcinoma; CCRT, concurrent chemoradiation therapy; NOS, Newcastle–Ottawa Scale; RCT, randomized controlled trial; SCC, squamous cell carcinoma.

### Quality assessment

**Figure 2:**
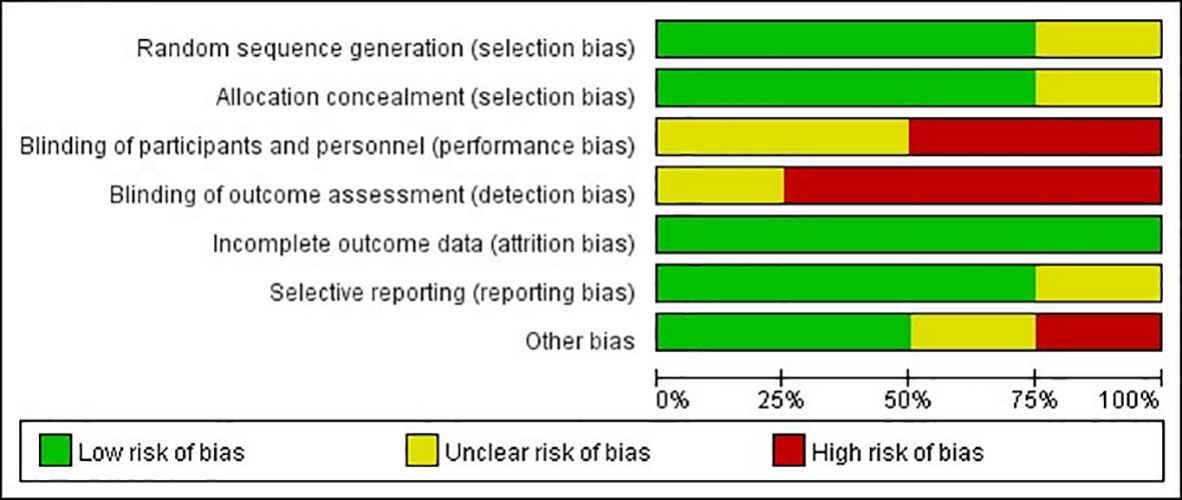
Risk of bias graph: review authors’ judgments about each risk of bias item presented as percentages across RCTs.

**Figure 3:**
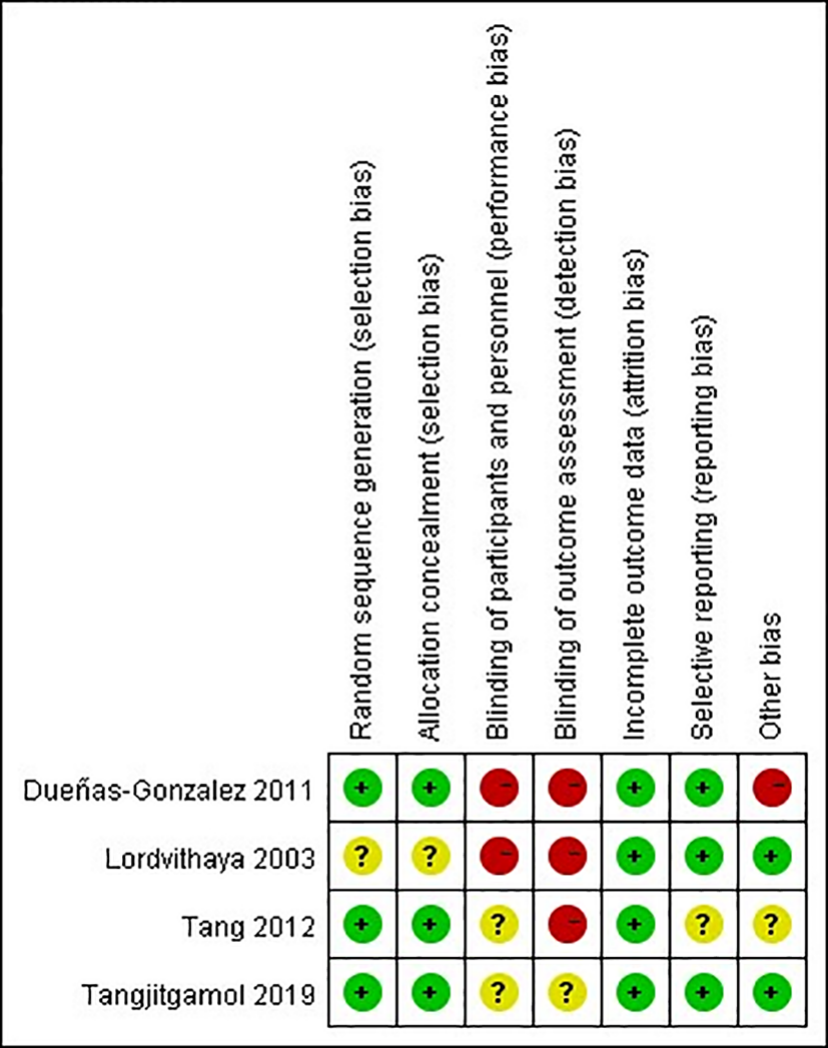
Risk of bias summary: review authors’ judgments about each risk of bias item for RCTs.

The quality of observational studies was determined using the Newcastle-Ottawa Scale. Any study that scored over seven stars was regarded as a high-quality study, while a score of four to six stars was regarded as a moderate-quality study (33). A quality assessment of the RCTs was carried out using the Cochrane risk of bias tool (**Figures 2**, **3**). High risk was mainly attributed to blinding methods. Most studies had either low or unclear risks of bias due to missing information on the protocols of the trials or inclusion criteria.

### Sensitivity analysis

**Figure 4:**
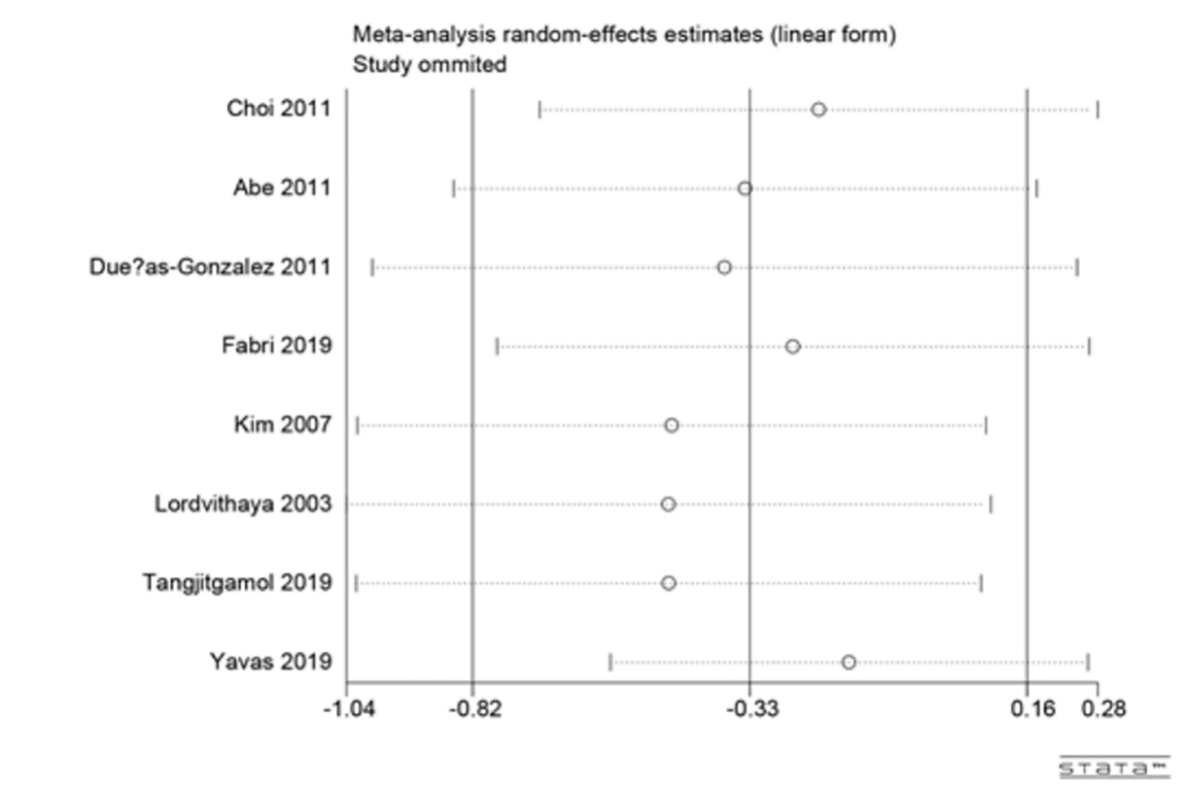
Sensitivity analysis of the 3-year rates of OS.

Large heterogeneity was observed among studies in this meta-analysis. Thus, we conducted sensitivity analyses for the 3-year rates of overall survival (OS) to explore the underlying reasons for heterogeneity (**Figure 4**). The pooled HR did not change significantly after sensitivity analysis with the removal of one study at a time, which indicated that the results were relatively stable.

### Primary endpoints: 3-year OS and progression-free survival

**Figure 5:**
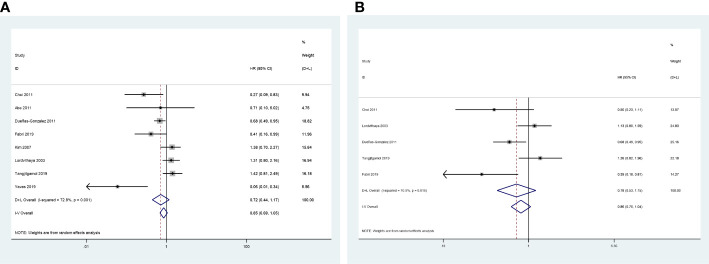
Forest plot of the survival rates. **(A)** 3-year rates of OS; **(B)** 3-year rates of PFS.

The 3-year OS was evaluated in eight included studies (24, 25, 27–32), and significant heterogeneity was observed among the studies (*P* = 0.001, *I^2^* = 72.8%). No significant difference was observed between the CCRT group and the CCRT plus ACT group (HR = 0.72, 95%CI = 0.44–1.17) (**Figure 5A**).

Five studies reported the HR for 3-year progression-free survival (PFS) (25, 27–29, 31). The results revealed no significant difference in 3-year PFS between the two groups (HR = 0.78, 95%CI = 0.53–1.15), with high level of heterogeneity between studies (*P* = 0.010, *I^2^* = 70.0%) (**Figure 5B**).

### Secondary endpoints: complete response, local recurrences, distant metastases, and adverse events

**Figure 6:**
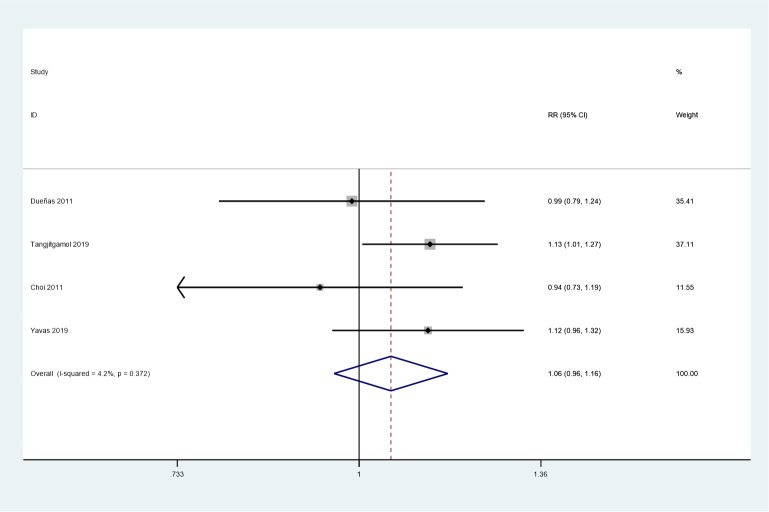
Forest plot of the complete response rate.

Four studies were included in this meta-analysis, which assessed the complete response rate (24, 25, 27, 28). No heterogeneity was observed among the studies (*P* = 0.372, *I^2^* = 4.2%). No noticeable differences were observed between the two groups in the complete response rate (RR = 1.06, 95%CI = 0.96–1.16) using a fixed-effects model. (**Figure 6**).

**Figure 7:**
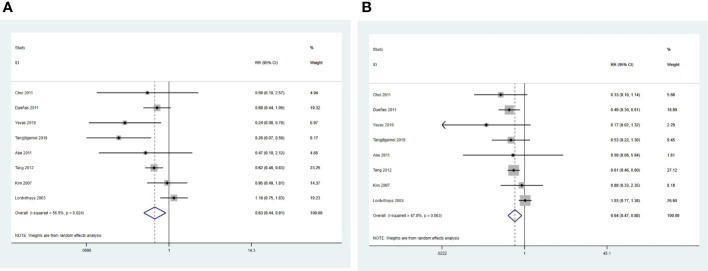
Forest plot of total failure: **(A)** local recurrences; **(B)** distant metastases.

Eight studies were pooled into the analysis of local recurrence rates (24–30, 32). The results indicated that the CCRT plus ACT group had a significantly lower risk of local recurrence than the CCRT group (RR = 0.63, 95%CI = 0.44 –0.91) with moderate between study heterogeneity (*P* = 0.024, *I^2^* = 56.5%) (**Figure 7A**).

Eight studies were eligible to analyze the risk of distant metastasis (24–30, 32). The results suggested that the risk of distant metastasis was significantly lower in the CCRT plus ACT group than in the CCRT group (RR = 0.64, 95%CI = 0.47–0.88) with moderate heterogeneity (*P* = 0.063, *I^2^* = 47.8%) between studies (**Figure 7B**).

**Figure 8:**
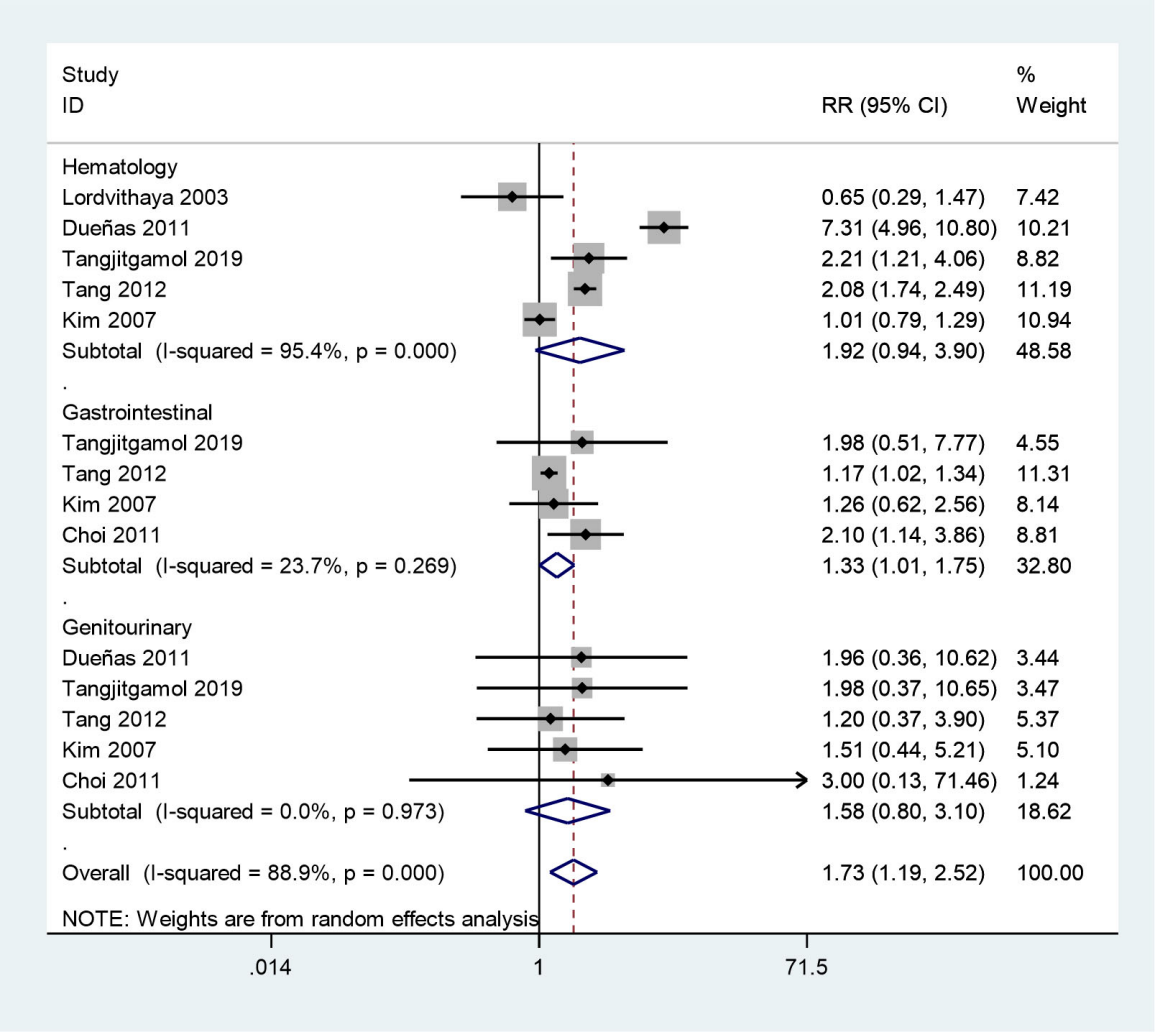
Forest plot of adverse events.

Six studies reported grade 3–4 acute toxicities in two groups (25–30). The meta-analysis showed that grade 3–4 acute toxicities were more frequent in the CCRT plus ACT group (RR = 1.73, 95%CI = 1.19–2.52) with high between study heterogeneity (*P* = 0.001, *I^2^* = 88.9%) (**Figure 8**). Next, we conducted a subgroup analysis, in which grade 3–4 gastrointestinal system toxicities were more frequent during the treatment of the CCRT plus ACT group (RR = 1.33, 95%CI = 1.01–1.75). However, no noticeable differences were observed between the two groups in grade 3–4 hematological adverse events (RR = 1.92, 95%CI = 0.94–3.90) and genitourinary system toxicities (RR = 1.58, 95%CI = 0.80–3.10).

### Publication bias

**Figure 9:**
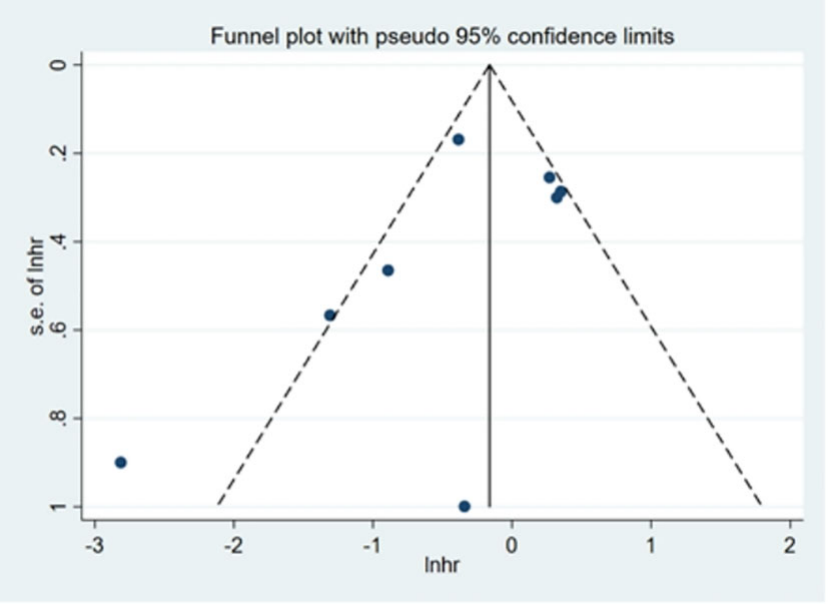
Begg’s funnel plot for detecting publication bias.

Visual inspection of Begg’s funnel plot did not identify substantial asymmetry (**Figure 9**). The publication bias was examined using Egger’s (*P* = 0.289) and Begg’s tests (*P* = 0.266), and no publication bias was found.

## Discussion

In 2021, the American Society of Clinical Oncology announced the latest results of the OUTBACK trial (34), which indicated that the addition of ACT to standard CCRT did not improve the survival outcomes of patients with LACC, and the incidence of adverse events was higher. This was a phase III multi-center clinical study with patients from developed countries, such as the United States and Canada. However, due to the limited medical facilities and detection capacities in developing countries, the incidence of LACC is higher. Most of the included studies (6/9) in this meta-analysis were from developing countries. This is the first meta-analysis aimed at comparing the tumor response, survival benefit, and tolerability between the CCRT plus ACT and CCRT-alone groups for patients with LACC. The results revealed that CCRT plus ACT was associated with the reduced risk of local recurrence and distant metastasis, yet at the expense of some additional toxicities. Nevertheless, the addition of ACT had no advantage in increasing the survival and complete response rates.

In recent years, ACT has been applied in different types of tumors, and its efficacy has been confirmed. In contrast, accurate data on the effects of ACT when added to CCRT in patients with LACC, still remain unclear (35). Despite the benefits of ACT reported in a number of previous studies (36–38), we failed to find any improvement in the survival or the complete response rate with increasing the incidences rates of grade 3–4 acute toxicities.

Reviews exploring the role of ACT after CCRT in patients with LACC have been limited. A 2021 systematic review did not demonstrate the effectiveness of ACT because the purpose of this review was to emphasize the importance of adjuvant systemic treatment (chemotherapy, immunotherapy, and hormone therapy) (39). Moreover, the control group in this review was not the CCRT-alone group. Because of the significant clinical differences between the included studies, no meta-analysis was conducted in the 2014 Cochrane review (9). This review only incorporated two RCTs, and one of the trials did not use platinum-based chemotherapy as adjuvant chemotherapy. Besides this, in the Cochrane review, concurrent chemotherapy regimens were not the same in the treatment group (gemcitabine plus cisplatin) and the control group (cisplatin). Thus, some limitations were found in applying their results to guide the application of ACT in clinical practice. This meta-analysis included more high-quality RCTs and other original studies, and provided more powerful and reliable results compared with the two previous studies.

It is noteworthy that three additional studies also investigated the role of ACT in patients with LACC. Jelavić et al. reported that ACT consisting of four cycles of cisplatin and ifosfamide after CCRT could potentially improve distant control of LACC (40). Mabuchi et al. observed that using three cycles of ACT with paclitaxel plus carboplatin after CCRT in patients with LACC of stage IIIB/IVA improved local control and reduced distant metastasis (36). However, the OUTBACK trial showed that four cycles of carboplatin combined with paclitaxel after concurrent chemoradiotherapy did not differ in local recurrence and distant metastasis compared with concurrent chemoradiotherapy alone (34). This meta-analysis found that local recurrence and distant metastasis were significantly lower in the CCRT plus ACT than in the CCRT group (RR = 0.63, 95%CI = 0.44 –0.91 and RR = 0.64, 95%CI = 0.47–0.88). One of the reasons might be that the systemic cytotoxic effects of ACT are enhanced by CCRT due to radiosensitization, rather than the effects of ACT alone (40).

The overdiagnosis and overtreatment of a malignant tumor is a serious issue and has been debated globally over the last few years (41). In principle, it should be emphasized that the superior treatment effect can be achieved only if moderate treatment is adopted. Overtreatment results in the waste of resources and places patients at risk of adverse events. For example, in this meta-analysis, grade 3–4 gastrointestinal system toxicities were found more frequent during the treatment of CCRT plus ACT than that of CCRT alone. Moreover, the total incidence of grade 3–4 adverse advents was more common in the CCRT plus ACT group than in the CCRT-alone group. Furthermore, ACT could not improve the survival rates in LACC, and therefore, ACT could be considered overtreatment. Multiple factors that might affect therapeutic options in patients with LACC should be taken into consideration when clinicians determine the appropriate therapeutic regimen to avoid overtreatment.

The treatment of patients with LACC has been under investigation. According to the National Comprehensive Cancer Network clinical guidelines, CCRT is still the preferred treatment option for stage IB3 and IIA2 cervical cancer, followed by radical hysterectomy combined with pelvic lymphadenectomy (42). For stage IIB cervical cancer, CCRT remains the only option (42). However, radiotherapy can impair the ovarian function and vaginal elasticity in young patients and reduce the quality of their sexual life (43). In recent years, some studies have shown that radical surgery after neoadjuvant chemotherapy can be an important treatment option for patients with LACC, and may have better performance than CCRT, especially in relatively early-stage patients (44–46).

This meta-analysis had several limitations. First, about half of the included studies (5/9) were observational, indicating that recalling bias and selection bias were hard to avoid. Second, in this meta-analysis, some survival outcomes extracted from the Kaplan–Meier curve might not accurately reflect the true values. Third, the ACT regimens differed slightly between studies; Lorvidhaya (2003) used non-platinum regimens (29). Fourth, only 2732 patients were included in trials, and the sample size in this meta-analysis needed to be further expanded. Fifth, the loss to follow-up in these studies might affect the results. Although most of the loss to follow-up in the four RCTs and five observational studies were balanced across treatment arms, the risk of selection bias could not be completely ignored, and the individuals who participated in these studies might not be representative of the randomized sample. Sixth, the length of the follow-up time of the included studies was relatively short. Finally, a large heterogeneity was observed in this study. However, the sensitivity analysis showed that the results were relatively reliable. The causes of heterogeneity might be different follow‐up periods, small sample size, different study designs, and different chemotherapy regimens.

## Conclusions

Compared with CCRT, ACT did not significantly improve OS and PFS rates with increasing unmanageable toxicity in the treatment of patients with LACC. The CCRT plus ACT treatment should not be considered over CCRT alone for LACC. Future studies need consideration of higher-quality RCTs to confirm this result.

## Data availability statement

The original contributions presented in the study are included in the article/Supplementary Material. Further inquiries can be directed to the corresponding authors.

## Author contributions

HNL, XBQ and ZXH contributed to conception and design of the study. CYS, NY and MW collected and assessed the literature. HNL, SZ and ZYX performed the statistical analysis. HNL and SYL wrote the first draft of the manuscript. All authors contributed to the article and approved the submitted version.
